# Prediction of Small for Gestational Age Infants in Healthy Nulliparous Women Using Clinical and Ultrasound Risk Factors Combined with Early Pregnancy Biomarkers

**DOI:** 10.1371/journal.pone.0169311

**Published:** 2017-01-09

**Authors:** Lesley M. E. McCowan, John M. D. Thompson, Rennae S. Taylor, Philip N. Baker, Robyn A. North, Lucilla Poston, Claire T. Roberts, Nigel A. B. Simpson, James J. Walker, Jenny Myers, Louise C. Kenny, On behalf of the SCOPE consortium

**Affiliations:** 1 Department of Obstetrics and Gynaecology, University of Auckland, Auckland, New Zealand; 2 South Auckland Clinical School, Middlemore Hospital, Department of Paediatrics: Child and Youth Health, University of Auckland, Auckland, New Zealand; 3 National Women’s, Auckland District Health Board, Auckland, New Zealand; 4 Department of Paediatrics, University of Auckland, Auckland, New Zealand; 5 Liggins Institute, University of Auckland, Auckland, New Zealand; 6 Division of Women’s Health, Women’s Health Academic Centre, King’s College London and King’s Health Partners, London, United Kingdom; 7 Robinson Research Institute and School of Medicine, University of Adelaide, Adelaide, Australia; 8 Section of Obstetrics and Gynaecology, Leeds Institute of Biomedical & Clinical Sciences, University of Leeds, Leeds, United Kingdom; 9 Maternal and Fetal Heath Research Centre, University of Manchester, Manchester, United Kingdom; 10 INFANT (The Irish Centre for Fetal and Neonatal Translational Research) Department of Obstetrics and Gynaecology, University College, Cork, Ireland; 11 The Department of Obstetrics and Gynaecology, University College Cork, Ireland; Stellenbosch University, SOUTH AFRICA

## Abstract

**Objective:**

Most small for gestational age pregnancies are unrecognised before birth, resulting in substantial avoidable perinatal mortality and morbidity. Our objective was to develop multivariable prediction models for small for gestational age combining clinical risk factors and biomarkers at 15±1 weeks’ with ultrasound parameters at 20±1 weeks’ gestation.

**Methods:**

Data from 5606 participants in the Screening for Pregnancy Endpoints (SCOPE) cohort study were divided into Training (n = 3735) and Validation datasets (n = 1871). The primary outcomes were All-SGA (small for gestational age with birthweight <10th customised centile), Normotensive-SGA (small for gestational age with a normotensive mother) and Hypertensive-SGA (small for gestational age with an hypertensive mother). The comparison group comprised women without the respective small for gestational age phenotype. Multivariable analysis was performed using stepwise logistic regression beginning with clinical variables, and subsequent additions of biomarker and then ultrasound (biometry and Doppler) variables. Model performance was assessed in Training and Validation datasets by calculating area under the curve.

**Results:**

633 (11.2%) infants were All-SGA, 465(8.2%) Normotensive-SGA and 168 (3%) Hypertensive-SGA. Area under the curve (95% Confidence Intervals) for All-SGA using 15±1 weeks’ clinical variables, 15±1 weeks’ clinical+ biomarker variables and clinical + biomarkers + biometry /Doppler at 20±1 weeks’ were: 0.63 (0.59–0.67), 0.64 (0.60–0.68) and 0.69 (0.66–0.73) respectively in the Validation dataset; Normotensive-SGA results were similar: 0.61 (0.57–0.66), 0.61 (0.56–0.66) and 0.68 (0.64–0.73) with small increases in performance in the Training datasets. Area under the curve (95% Confidence Intervals) for Hypertensive-SGA were: 0.76 (0.70–0.82), 0.80 (0.75–0.86) and 0.84 (0.78–0.89) with minimal change in the Training datasets.

**Conclusion:**

Models for prediction of small for gestational age, which combine biomarkers, clinical and ultrasound data from a cohort of low-risk nulliparous women achieved modest performance. Incorporation of biomarkers into the models resulted in no improvement in performance of prediction of All-SGA and Normotensive-SGA but a small improvement in prediction of Hypertensive-SGA. Our models currently have insufficient reliability for application in clinical practice however, they have potential utility in two-staged screening tests which include third trimester biomarkers and or fetal biometry.

## Introduction

Approximately 40% of non-anomalous singleton stillbirths are small for gestational age (SGA) [1, 2] and live born SGA infants have increased risk of long-term adverse outcomes.[3–5] Placental insufficiency is a major contributor to the pathophysiology in SGA pregnancies.[6]

A limitation of antenatal care is that the majority of SGA pregnancies are not identified before birth.[7–9] SGA infants recognized before birth and delivered in a timely fashion have a four-fold reduction in composite severe morbidity/ mortality.[10] Reliable early pregnancy risk prediction, therefore has potential to reduce morbidity and mortality. As we have previously reported,[11] SGA infants can be broadly classified into two categories with distinct maternal phenotypes: SGA with a normotensive mother (Normotensive-SGA) and SGA where the mother has gestational hypertension, preeclampsia or chronic hypertension (Hypertensive-SGA).[7] We have previously reported that Normotensive- SGA comprise approximately three quarters of SGA infants and that risk factors for Normotensive-SGA and Hypertensive-SGA differ, suggesting they are distinct conditions from the prediction perspective.[12] We have recently published risk prediction models for these SGA sub-groups, derived from participants in the Screening for Pregnancy Endpoints (SCOPE) study, combining early pregnancy clinical variables with ultrasound parameters from the 20±1 weeks’ anatomy scan. Only modest predictive performance was achieved.[11] Abnormal placentation may be detected by altered biomarker concentrations in early pregnancy.[13–16] A recent systematic review of first trimester biomarkers to predict SGA reported that biomarkers alone had low predictive accuracy but speculated that performance would improve with addition of clinical characteristics and uterine artery Doppler.[17] An increase in predictive performance for SGA has been reported, after addition of first trimester biomarkers and uterine artery Doppler to clinical risk factors.[18]

Our primary objective was to develop multivariable prediction models for the respective SGA phenotypes, by combining biomarkers with clinical risk factors measured at 15±1 weeks’ and with uterine artery Doppler indices and fetal biometry at 20±1 weeks’ gestation. Since customized birthweight centiles may better identify small vulnerable babies with placental dysfunction, we used customized centiles to define SGA.[19–21]

We hypothesised that addition of 15±1 weeks’ biomarker data to models comprising clinical and ultrasound variables would result in significant improvements in prediction of SGA pregnancies.

## Methods

The participants were healthy nulliparous women with singleton pregnancies recruited to the SCOPE study between November 2004 and February 2011 in Auckland, New Zealand, Adelaide, Australia, Manchester, Leeds and London, United Kingdom and Cork, Ireland. SCOPE (www.scopestudy.net) is a prospective, multi-centre cohort study with the main aim of developing screening tests to predict preeclampsia, SGA infants and spontaneous preterm birth. Ethical approval was obtained from institutional ethics committees of each participating center and all women provided written informed consent [New Zealand AKX/02/00/364–23 April 2003; Australia REC 1712/5/2008–2 November 2005; London and Manchester 06/MRE01/98–19 January 2007; Leeds 06/MRE01/98–5 November 2007 and Cork ECM5 (10) 05/02/08–6 February 2008]. Detailed methods have previously been described.[7, 22]

Exclusion criteria included 1) an elevated risk of preeclampsia, small for gestational age (SGA) or spontaneous preterm birth due to underlying medical conditions (known chronic hypertension, and/or pre-existing diabetes, renal disease, systemic lupus erythematosus, or anti-phospholipid syndrome), previous cervical knife cone biopsy, ≥3 terminations or ≥3 miscarriages or current ruptured membranes; 2) known major fetal anomaly or abnormal karyotype or 3) interventions (such as low dose aspirin) that might modify pregnancy outcome.[23] Women were recruited at 15±1 weeks’ gestation. Of the 8531 women invited to participate 5989 (70%) agreed and were interviewed and examined by a research midwife at 15±1 and 20±1 weeks’ and underwent ultrasound examination at 20±1 weeks’. Detailed clinical data were collected at each time point. The estimated date of delivery was calculated as follows: if the woman had a certain last menstrual period (LMP) date, the estimated date of delivery was only adjusted if either 1) a scan performed at <16 weeks’ gestation found a difference of ≥7 days between the scan gestation and that calculated by the LMP or 2) on 20±1 week scan a difference of ≥10 days was found between the scan gestation and that calculated from the LMP. If her LMP date was uncertain, then scan dates were used to calculate the estimated date of delivery. At the interview, data were entered into a secure internet-accessed, auditable database (MedSciNet AB, Sweden).

Ultrasound examination at 20±1 weeks’ was performed by trained sonographers and included fetal biometry and Doppler studies of the umbilical and uterine arteries. Fetal measurements were adjusted for gestational age by calculating the multiple of the median for each gestational week. Mean uterine resistance index (RI) was calculated from the left and right uterine RI. If only a left or right uterine RI was available, this was used as ‘mean RI’ (n = 98). Women without fetal biometry or Doppler studies were excluded from the analysis of ultrasound factors. Participants were followed prospectively, with pregnancy outcome data and infant measurements recorded by research midwives, usually within 72 hours of birth.

### Outcome measures

*All-SGA* was defined as birthweight <10^th^ customised centile, adjusted for maternal height, booking weight, ethnicity, delivery gestation and infant sex.[24] *Normotensive-SGA* was defined as birth of an SGA infant where the mother did not develop hypertension, and *Hypertensive-SGA* defined as birth of an SGA infant where the mother had developed gestational hypertension or preeclampsia and/or had exhibited mild chronic hypertension.^7^

### Definitions

*Gestational hypertension*: systolic BP≥140 mmHg and/or diastolic BP ≥90mmHg on at least 2 occasions 4 hours apart after 20 weeks’, before the onset of labour. *Preeclampsia*: gestational hypertension or postpartum hypertension (as defined above, but developing for the first time after delivery) in association with proteinuria (24 hour urinary protein ≥ 300 mg, or spot urine protein: creatinine ratio ≥ 30 mg/mmol, or urine dipstick protein ≥ 2+) or any multi-system complication of preeclampsia.[23, 25] *Mild chronic hypertension*: systolic BP of 140–159 mmHg and/or diastolic BP 90–99 mmHg, on more than one reading, first identified at the 15±1 or 20±1 weeks’ SCOPE visit. No participants had recognised or treated hypertension before pregnancy. *Non-SGA* referred to all women who did not have SGA babies. This group included pregnancies with other complications such as spontaneous preterm birth or preeclampsia without SGA.

### Datasets

To allow for model generalization, data was partitioned into a Training set for model fitting and a Validation set for empirical validation. A 2:1 ratio was achieved by randomly splitting the total dataset stratified by geographical areas of Australasia and Europe. The datasets were checked for major discrepancies in SCOPE centre and rates of SGA.

### Clinical and ultrasound variables

Details of clinical and ultrasound variable selection and the variable reduction process have previously been reported.[11] Details of clinical variables used in the SGA models are available as supplementary material (Table A in S1 Appendix).

### Biomarkers

Fifty three biomarkers were selected based on either *à priori* knowledge of a biological role in: i) placentation, ii) angiogenesis or inflammation, iii) an association with preeclampsia, or iv) involvement in glucose or lipid metabolism. A full list of all but seven of the biomarkers, details of ELISA methodologies and biomarker data transformation methodology has previously been published.[26] The additional seven biomarkers included adiponectin, total cholesterol, HDL-cholesterol, insulin, LDL-cholesterol, human placental growth hormone, and triglycerides. Details of these biomarkers and ELISA methodologies are included as supplementary material (S1 Appendix and Table B in S1 Appendix).

### Statistical analysis

SAS (version 9.3) Cary, N.C. was used for statistical analyses. The comparison group for All-SGA was Not-SGA, for ‘Normotensive-SGA’ was ‘Not Normotensive-SGA’ (all women who did not have ‘Normotensive-SGA’ including those with ‘Hypertensive-SGA’), and for ‘Hypertensive-SGA’ the comparison group was ‘Not Hypertensive-SGA’ (all women who did not have ‘Hypertensive—SGA’ including those with ‘Normotensive-SGA’). Comparison of population characteristics and pregnancy outcomes was performed using Student’s *t*-test, analysis of variance (ANOVA), the Wilcoxon Rank Sum test or the χ^2^ test, as appropriate for data type. Comparisons of biomarker distributions were performed using t-tests on the log-transformed data, with p-values adjusted to control the False Discovery Rate.[27] Single variable logistic regression was then applied to each log-transformed biomarker, along with calculation of the Area under the Receiver Operating Characteristic curve (AUC).

### Multivariable modelling

Single variable logistic regression for each SGA endpoint was used in the Training cohort to select biomarkers for potential inclusion in multivariable predictive models. A significance threshold for the adjusted p-values of 0.05 was used for variable selection. Distinct models were constructed per endpoint (All-SGA, Normotensive-SGA and Hypertensive-SGA) as follows: (1) biomarkers only measured at 15±1 weeks’ (2) clinical risk factors at 15±1 weeks’ (3) combination of biomarkers and clinical risk factors at 15±1 weeks’ (4) combinations of clinical risk factors (15±1 weeks’), biomarkers (15±1 weeks’) and ultrasound variables (20±1 weeks’).

The solely biomarker and clinical risk factor logistic regression models were constructed using backwards stepwise variable selection, starting from a model containing all available biomarker or clinical variables, respectively. Only biomarkers included in the ‘biomarker only’ model for the SGA endpoint were available for the combined logistic regression models. Similarly, only variables that remained in the multivariable logistic regression model using ‘clinical risk factors only’ for the SGA endpoint were available to the ‘combined biomarker and clinical risk factor’ models. Again, logistic regression models were constructed using backwards stepwise variable selection. Model performance was assessed in the Training and Validation datasets via calculation of AUC for each model, along with sensitivity, positive and negative predictive value, and positive and negative likelihood ratios at a 5% false positive rate (95% specificity).

## Results

**Fig 1:**
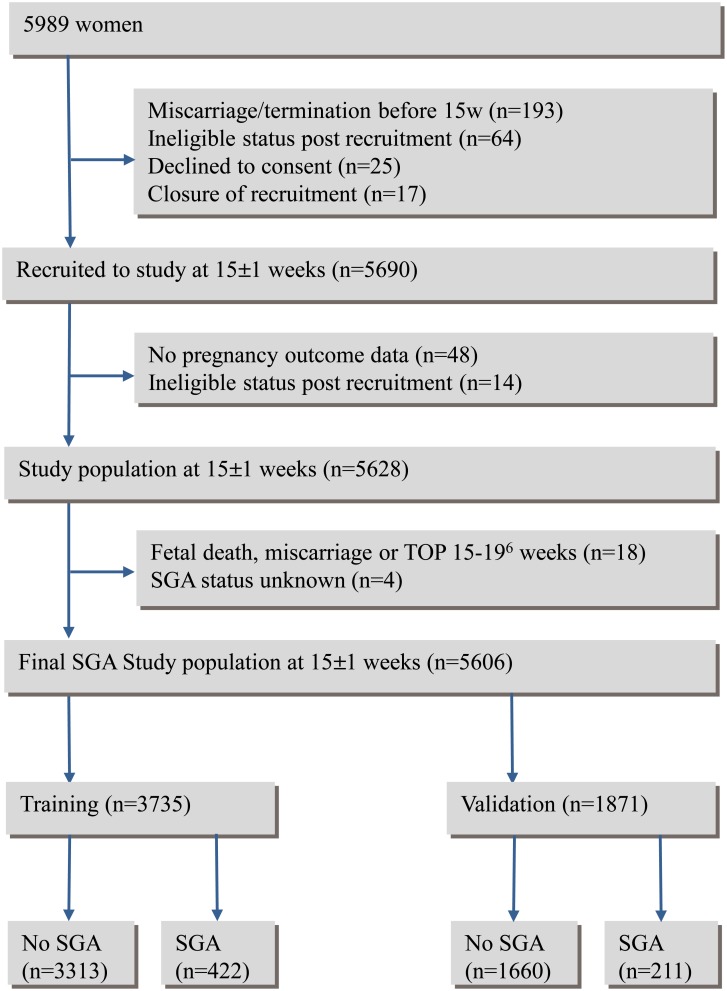
Flow chart of study population.

Consistent with our previous report, [11] 5690 healthy nulliparous women were recruited between November 2004 and February 2011 in Auckland, New Zealand, Adelaide, Australia, London, Leeds and Manchester, United Kingdom and Cork, Ireland and follow up was complete in 98.9% of participants. Our final study population with SGA data available was 5606. (Fig 1).

The data were divided into a Training set for model fitting (n = 3735) and a Validation set (n = 1871) (Fig 1). Of the 422 (11%) infants in the Training database who were SGA by customized centiles (All-SGA), 313 (8%) were born to normotensive mothers (Normotensive-SGA) and 109 (3%) to hypertensive mothers (Hypertensive-SGA). In the Validation database, 211 (11%) infants were SGA of whom 152 (8%) were Normotensive-SGA and 59 (3%) Hypertensive-SGA. Comparing Training and Validation sets, amongst the Hypertensive-SGA pregnancies, 44 (40%) and 26 (44%) women respectively had preeclampsia; 63 (58%), and 33 (56%) had gestational hypertension; 2 (2%) and none had mild chronic hypertension alone.

The baseline characteristics and pregnancy outcomes of the women who had SGA infants are compared with those with non-SGA infants in Table 1. Women with SGA infants were less likely to be primigravid, more likely to be single, less likely to be employed, more likely to have lower socioeconomic status, twice as likely to smoke and less likely to have a normal BMI than women with non-SGA pregnancies. They also reported lower mean birthweight and had higher systolic and diastolic blood pressures at 15±1 weeks’. They had a threefold increase in preterm delivery and higher rates of perinatal death than mothers of non-SGA infants.

**Table 1:** Study Population Characteristics at 15 ±1 Weeks’ and Pregnancy Outcome in the Total SGA Cohort (N = 5606).

	Non-SGA	All-SGA	*p*-value
	N = 4973	N = 633	
**Maternal Characteristics**			
Ethnicity			0.54
• Caucasian	4481 (90)	564 (89)	
• Maori or Pacific Islander*	100 (2)	13 (2)	
• Indian	115 (2)	19 (3)	
• Asian	152 (3)	16 (3)	
• Other^†^	125 (3)	21 (3)	
Primigravid	3853 (78)	466 (74)	0.03
Single	458 (9)	79 (13)	0.009
No paid employment	708 (14)	118 (19)	0.003
<12 years education	1858 (37)	258 (41)	0.10
Smoking status at 15 wks			<0.0001
• Non smoker	3821 (77)	428 (68)	
• Ceased smoking before15wks	669 (13)	84 (13)	
• Current smoker	483 (10)	121 (19)	
BMI category			0.002
• <20.0	352 (7)	50 (8)	
• 20.0–24.9	2518 (51)	276 (44)	
• 25.0–29.9	1386 (28)	186 (29)	
• ≥30	717 (14)	121 (19)	
**Maternal Characteristics**			
BMI (kg/m^2^)	25.2 (4.8)	25.9 (5.5)	0.004
Maternal age (y)	28.7 (5.5)	28.6 (5.8)	.67
Socio-economic index	42 (17)	40 (16)	.006
Maternal birthweight (g)	3157 (908)	2960 (946)	<.0001
Systolic BP (mmHg)	107 (10)	108 (11)	<.0001
Diastolic BP (mmHg)	65 (8)	66 (9)	.0002
**Pregnancy Outcome**			
Birthweight (g)	3504 (505)	2609 (578)	<.0001
Gestational age at delivery (wks)	39.8 (1.9)	38.8 (3.5)	<.0001
Total preterm births (<37wks)	257 (5)	94 (15) ^‡^	<.0001
Admitted to neonatal unit	504 (10)	143 (23)	<.0001
Perinatal deaths^§^	16 (0.3)	12 (2)	<.0001
Hypertensive Pregnancy^¶^	592 (12)	168 (27)	<.0001

Results expressed as N (%) or mean (SD) as appropriate;

* includes 71 Maori and 42 Pacific Islanders;

^†^ includes 23 Australian Aborigines.

^‡^ 41 (8.8%) of Normotensive-SGA and 53 (31.5%) of Hypertensive-SGA

^§^ Rate/1000 births

^¶^ Hypertensive pregnancy defined as preeclampsia, gestational hypertension or mild chronic hypertension

Mean plasma biomarker concentrations (measured at 15±1 weeks’), results of univariable and multivariable analyses and AUCs for those biomarkers significantly associated with SGA, and the sub-phenotypes of SGA, are shown in Table 2. For All-SGA, eight biomarkers were associated with SGA after standard univariable analysis, with five remaining associated after controlling for the False Discovery Rate. The AUCs for these individual biomarkers was poor. After multivariable analysis, using Wilcoxon Rank Sum test, with p-values adjusted to control the False Discovery Rate, only two remained significantly associated with All-SGA in the reduced model: pregnancy-associated plasma protein A (PAPP-A) and vascular endothelial growth factor receptor-1 (VEGFR1) with AUC 0.60 (0.57–0.63).

**Table 2:** Univariable Analysis and AUC of Individual Biomarkers Measured at 15 ± 1 Weeks’ Gestation Comparing SGA Groups with Respective Controls.

Biomarker	Controls	SGA Group	*p*-value*	*q*-value^†^	Univariable AUC (95% CI)	Reduced
						Multivariable Model
						*p*-value
**ALL-SGA**						
Cystatin C, mg/mL	1873 (1501–2343)	1888 (1500–2461)	**.01**	.08	0.52 (0.49–0.55)	
MIF, ng/mL	9.2 (8.0–11.6)	9.0 (7.7–10.9)	**.002**	**.02**	0.54 (0.51–0.57)	
PAI-2^‡^, ng/mL	1.0 (0.8–1.3)	0.95 (0.7–1.2)	**.003**	**.02**	0.55 (0.52–0.58)	
PAPP-A^‡^, ng/mL	1.0 (0.6–1.8)	0.8 (0.5–1.5)	**<.001**	**.002**	0.58 (0.55–0.61)	**0.004**
PlGF^‡^, ng/mL	1.0 (0.6–1.7)	0.95 (0.5–1.7)	**.009**	.06	0.54 (0.51–0.57)	
VEGFR1, ng/mL	0.34 (0.21–0.54)	0.28 (0.18–0.45)	**<.001**	**.002**	0.58 (0.55–0.61)	**0.021**
Insulin, ng/mL	16.5 (9.7–27.7)	15.1 (8.7–25.5)	**.009**	.06	0.53 (0.50–0.56)	
Triglycerides^‡^, mg/dL	0.99 (0.8–1.2)	1.02 (0.8–1.3)	**<.001**	**.002**	0.52 (0.49–0.55)	
						**AUC = 0.59 (0.56–0.62)**
**NORMOTENSIVE-SGA**						
Caspase 3, ng/mL	2.4 (1.4–4.2)	2.0 (1.2–3.4)	**.001**	**.023**	0.56 (0.53–0.59)	**0.045**
MIF, ng/mL	9.2 (7.9–11.6)	9.0 (7-7-10.9)	**.004**	.06	0.54 (50.0–0.57)	
PAPP-A^‡^, ng/mL	1.0 (0.6–1.8)	0.8 (0.5–1.6)	**.0001**	**.003**	0.57 (0.53–0.60)	**0.032**
VEGFR1, ng/mL	0.27 (0.18–0.54)	0.34 (0.21–0.42)	**<.0001**	**.003**	0.59 (0.55–0.62)	**0.008**
						**AUC = 0.60 (0.57–0.63)**
**HYPERTENSIVE-SGA**						
PAPP-A^‡^, ng/mL	1.0 (0.6–1.8)	0.7 (0.4–1.5)	**.001**	**.006**	0.59 (0.53–0.65)	**0.025**
PlGF^‡^, ng/mL	1.0 (0.6–1.7)	0.7 (0.3–1.3)	**<.0001**	**.002**	0.60 (0.54–0.66)	**0.001**
Triglycerides^‡^, mg/dL	1.0 (0.8–1.3)	1.1 (0.9–1.5)	**<.0001**	**.002**	0.61 (0.55–0.67)	**0.0001**
						**AUC = 0.66 (0.60–0.71)**

AUC, area under the receiver operating curve; CI, confidence interval; MIF, macrophage migration inhibitory factor; PAI-2, plasminogen activator inhibitor 2; PAPP-A, pregnancy-associated plasma protein A; PlGF, placental growth factor; VEGFR, vascular endothelial growth factor receptor;

Biomarker concentrations are shown as median (interquartile range);

* Based on Analysis of Log Transformed Data

^†^ Based on False Discovery Rate

^‡^ Based on MoM Data

For Normotensive-SGA, Caspase-3 remained significant in the multivariable model together with PAPP-A and VEGFR1 with an AUC of 0.60 (0.57–0.63). For Hypertensive-SGA, PAPP-A, PlGF and Triglycerides were significantly discriminatory in univariable analysis after adjustment for False Discovery Rate. All remained significant after multivariable analysis, with an AUC of 0.66 (0.60–0.71).

The addition of biomarker data to clinical risk factors led to a small increment in AUC for All-SGA (2% in Training and 1% in Validation datasets) with a further modest increase with addition of ultrasound characteristics (6% and 5% increment for Training and Validation datasets respectively) with a final AUC for the Training dataset of 0.74 (0.71–0.76) and Validation dataset of 0.69 (0.66–0.73). Small incremental increases in AUC occurred in the Normotensive-SGA and Hypertensive-SGA prediction models with the addition of biomarker and ultrasound variables to clinical data. Final AUCs (which included ultrasound variables) were similar for All-SGA and Normotensive-SGA with final AUCs for Hypertensive-SGA approximately 10% higher (Table 3).

**Table 3:** AUCs from Multivariable Models to Predict SGA, Normotensive-SGA and Hypertensive-SGA.

	All-SGA		Normotensive-SGA		Hypertensive-SGA	
	AUC (95% CI)		AUC (95% CI)		AUC (95% CI)	
Variables in Model	Training	Validation	Training	Validation	Training	Validation
15 week Clinical*	0.66 (0.64–0.69)	0.63 (0.59–0.67)	0.66 (0.63–0.70)	0.61 (0.57–0.66)	0.76 0.72–0.81)	0.76 (0.70–0.82)
15 week Clinical* +Biomarkers^†^	0.68 (0.65–0.71)	0.64 (0.60–0.68)	0.69 (0.66–0.72)	0.61 (0.56–0.66)	0.78 (0.73–0.82)	0.80 (0.75–0.86)
15 week Clinical* +Biomarkers^†^						
+20 week Ultrasound^‡^	0.74 (0.71–0.76)	0.69 (0.66–0.73)	0.73 (0.70–0.76)	0.68 (0.64–0.73)	0.82 (0.78–0.86)	0.84 (0.78–0.89)

AUC, area under the receiver operating curve; CI, confidence interval;

* Clinical: **All-SGA** (family history of coronary heart disease, maternal birthweight, >12 months to conceive, attending university, smoking, proteinuria, vigorous exercise, diastolic BP ≥80mmHg, recreational walking, Rhesus negative blood group, random glucose); **Normotensive-SGA**: (maternal birthweight, attending university, smoking, vigorous exercise, recreational walking, Rhesus negative blood group, random glucose): **Hypertensive-SGA**: (>12 months to conceive, family history of metabolic syndrome, family history of coronary heart disease, low fruit consumption, binge drinking, maternal birthweight, body mass index, systolic blood pressure ≥120mmHg, diastolic BP ≥80mmHg) (12)

^†^ Biomarkers: **All-SGA** (PAPP-A, pregnancy-associated plasma protein A; PlGF, placental growth factor); **Normotensive-SGA**: (Caspase 3; PAPP-A; VEGFR1, vascular endothelial growth factor receptor); **Hypertensive-SGA**: (PAPP-A; PlGF; Triglycerides)

^‡^ Ultrasound: **All-SGA and Normotensive-SGA**: (head and abdominal circumference, uterine resistance index); **Hypertensive-SGA**: (uterine resistance index)

Table 4 summarizes the screening test characteristics of the final models combining clinical, biomarker and ultrasound variables in the Validation and Training datasets for the SGA groups at a 5% false positive rate.

**Table 4:** Screening Test Characteristics, at 95% Specificity, for All-SGA, Normotensive-SGA and Hypertensive-SGA of the Multivariable Models Based on Combining Clinical Risk Factors, Biomarkers and Ultrasound Data.

Clinical Group	Pretest Prevalence	Sensitivity	Positive Predictive Value	Negative Predictive Value	PositiveLikelihood Ratio	Negative Likelihood Ratio
**All-SGA**						
**Training**	11.3%	21 (17, 25)	35 (29, 41)	91 (90, 92)	4.20 (3.29, 5.36)	0.83 (0.79, 0.87)
**Validation**		19 (14, 25)	32 (23, 40)	91 (89, 92)	3.86 (2.69, 5.53)	0.85 (0.79, 0.91)
**Normotensive-SGA**						
**Training**	8.4%	21 (17, 26)	28 (22, 34)	93 (92, 94)	4.22 (3.24, 5.52)	0.83 (0.78, 0.88)
**Validation**		16 (9, 22)	20 (13, 28)	93 (92, 94)	3.09 (1.98, 4.83)	0.89 (0.83, 0.96)
**Hypertensive-SGA**						
**Training**	2.9%	28 (19, 38)	13 (8, 18)	98 (98, 99)	7.02 (5.20, 9.49)	0.68 (0.59, 0.79)
**Validation**		42 (29, 55)	20 (13, 27)	98 (97, 99)	8.38 (5.81, 12.11)	0.61 (0.49, 0.76)

## Discussion

We present a series of models, developed in a large, well-phenotyped international pregnancy cohort of low-risk nulliparous women, which combine biomarkers, clinical and ultrasound data to predict the risk of SGA and its different phenotypes. We have previously performed a detailed analysis of early pregnancy clinical risk factors combined with ultrasound parameters at 20±1 weeks’ gestation in this cohort that resulted in modest performance for the prediction of SGA (AUC of 0.69 for All-SGA).[11] We hypothesized that early pregnancy prediction of SGA would be improved by addition of biomarkers, in line with previous studies.[18] However, despite the selection of the biomarkers for known associations, no single biomarker, or combination of biomarkers, substantially improved the performance of the clinical/ultrasound-based risk model. As the phenotypes of SGA have low prevalence (from 2%-14% of the antenatal population), a clinically useful test generally needs to have a high positive LR (> 10) and low negative LR (< 0.10).[28] The performance of our models for the prediction of All-SGA and its phenotypes in healthy nulliparous women, by these stringent criteria, is insufficient for application in clinical practice.

Interesting insights were provided into possible biological determinants of the different SGA phenotypes. PAPP-A, a reduction in which was common to all models, is a syncytiotrophoblast-derived metalloprotease, which binds to and cleaves insulin-like growth factor binding proteins IGFBP- 3 and IGFBP-4.[29] These binding proteins, and cleavage products, have reduced affinity for the IGFs, thereby increasing biologically available IGFs. This increases placental growth and function, which enhances nutrient transport to the fetus.[30, 31] Low PAPP-A, results in less bioavailable IGFs and decreased growth.[32] Our findings are consistent with several large population-based screening studies [33–35] and a recent meta-analysis.[17]

The majority of SGA infants (73.8%) were born to mothers who remained normotensive in pregnancy and hence there is substantial overlap between All-SGA and Normotensive-SGA clinical risk predictors [11] which is mirrored by the biomarker data. In addition to PAPP-A, raised VEGFR1 was common to All-SGA and Normotensive-SGA. VEGF is involved in vasculogenesis and angiogenesis in early placental development,[36] and has both membrane bound and circulating soluble forms (soluble fms-like tyrosine kinase-1 or sFlt-1). sFlt-1 binds and reduces free levels of VEGF, thereby blunting the pro-angiogenic effect reducing fetal growth.

The performance of each of our models, though comparable with reports by others,[37] was insufficient to warrant introduction into clinical practice. Additional information in later gestation may be necessary to improve prediction of risk for SGA. Arguably, the clinical need for an accurate screening test is greatest in Normotensive-SGA; unlike Hypertensive-SGA, these pregnancies often lack overt clinical signs to alert the clinician to the at-risk fetus.

Hypertensive-SGA comprised of 96/168 (57%) mothers with gestational hypertension and 70/168 (42%) with preeclampsia.[25] The group with gestational hypertension and SGA would now meet the updated criteria for definition of preeclampsia.[38] Overall, 31.5% of women with Hypertensive-SGA were delivered at < 37 weeks highlighting the combination of fetal and maternal manifestations of defective early placentation. In line with this, a reduction in placental growth factor (PlGF) was common to models for preeclampsia and Hypertensive-SGA.[26] PlGF, a member of the VEGF family, is an angiogenic, pro-inflammatory factor produced by trophoblast cells, with a central role in the regulation of VEGF–dependent angiogenesis.[39] Consistent with our results, recent evidence suggests that low PlGF has potential to be incorporated in screening for early-onset disease, which is often accompanied by uteroplacental dysfunction and SGA.[14, 26] Another interesting finding was the role of Caspase-3, important in apoptosis, in normotensive-SGA and not found in Hypertensive-SGA. Apoptosis is important in remodeling of developing tissue, including the placenta as well as pathological conditions.[40, 41] Its role in the etiology of placental development and disease needs to be explored.

Strengths of the study include the unique composition of the SCOPE cohort, which aimed to predict late pregnancy complications in low-risk nulliparous women who comprise approximately 40% of births in many Western countries.[42–45] The biobank was prospectively collected and curated using a rigorous protocol and pre-specified clinical phenotyping. Stronger prediction of SGA has been reported in some previous studies,[18,46] but these were typically conducted in heterogeneous populations including women with underlying medical conditions [18] or where the control population comprised women with uncomplicated pregnancies.[46] In both scenarios, screening performance can be overestimated.

Limitations include the ethnic homogeneity, more than 90% of participants being Caucasian. The low incidence of SGA subtypes (e.g. Hypertensive-SGA) may have led to models being over-fitted. Moreover, SCOPE’s primary aim [26] was to screen and select biomarkers for prediction of preeclampsia, and prediction of SGA was a planned secondary analysis. Other biomarkers for future consideration might include glycogen phosphorylase isoenzyme BB [47] or beta-hCG.[34,48] Metabolomic analyses warrant further investigation as we recently demonstrated in a case-control study that 19 metabolites combined to give an AUC for SGA of 0.9.[49] Finally, the SCOPE study was designed to generate early pregnancy screening tests and did not include a third-trimester blood sample or ultrasound scan. For early pregnancy prediction of SGA, our models are inadequate for clinical use. However, PlGF (and possibly other biomarkers) may have greater utility closer to disease onset [50–52] and there is potential for evaluation of a two-stage test with those who screen positive in early pregnancy receiving low dose aspirin [53] and late pregnancy screening with PlGF and/or a growth scan at 36 weeks.[54] Sovio et al [54] reported that 57% of SGA infants were identified by ultrasound performed in a research setting at 36 weeks of gestation whereas scanning earlier in the third trimester is less reliable for detection of SGA.[55] An algorithm whereby women who have moderate or high risk screening for SGA at 19–24 weeks are selectively offered further third trimester scanning at 32 and or 36 weeks has been proposed and now requires validation.[56]

## Conclusions

Modest prediction of SGA was obtained by combining early pregnancy clinical risk factors, biomarkers and biometry and Doppler data from the 20-weeks’ anatomy scan in healthy nulliparous women. Addition of biomarker data to our previous clinical and ultrasound models did not improve performance. There is an urgent need to develop reliable tools for prediction of SGA, especially the majority group of Normotensive-SGA, who are usually not detected until after birth. Such tools could reduce perinatal morbidity and mortality. This paper gives new insights into the potential molecular mechanisms of placental and hypertensive disease in pregnancy.

## Supporting Information

S1 Appendix (includes Table A and Table B). (DOCX)
