# Supplementary material for: Prediction of Small for Gestational Age Infants in Healthy Nulliparous Women Using Clinical and Ultrasound Risk Factors Combined with Early Pregnancy Biomarkers
Source: PLoS One. 2017 Jan 9;12(1):e0169311. doi: 10.1371/journal.pone.0169311 (PMC5221822; doi:10.1371/journal.pone.0169311)
Supplement: S1 Appendix — (DOCX) [file pone.0169311.s001.docx]

**S 1 Appendix:**

**Clinical and biochemical factors associated with small for gestational age**

**Methodology**

Plasma, collected in a BD 6ml plastic EDTA spray coated vacutainer tube, was centrifuged at 3000 rpm (2400xg) for 10 minutes at 4^Ο^ C and then aliquoted into 0.25ml barcoded aliquots and stored at -80°C within four hours of collection. Specimens were kept on ice during the transport and processing phase and no specimens went through a freeze/thaw cycle prior to analyses.

Of the 7 additional biomarkers assessed in this analysis, 3 (total cholesterol, HDL-cholesterol and triglycerides) were measured using Beckman Unicel Syncron DxC (Beckman Coulter, Inc., Brea, CA 92821 USA). Measurements were not repeated for participants with previously available results for these analytes, which were performed using COBAS (Roche Diagnostics Ltd., CH-6343 Rotkreuz, Switzerland).^1^ Both methods used the same reactions. The results from the Beckmann Unicel Syncron DxC and the Roche Cobas platforms were compared. This analysis showed small differences in mean lipid values. However after adjusting for factors such as BMI and age which are related to lipid levels and also between study centres the differences between platforms were minimal. This suggests any differences are largely explained by population characteristics rather than the testing platforms. As an additional check the univariable relationships of the lipids stratified by platform were investigated and the results are consistent.

The LDL-cholesterol was calculated using the Friedewald Formula. The remaining three additional biomarkers (adiponectin, insulin and placental growth hormone) were measured using the same platform used for measurement of the 46 previously reported biomarkers^2^ with the methodology below.

Mouse derived recombinant Fab(s), generated by unique phage display technology (Alere, San Diego), were used in several immunoassay formats for the measurement of biomarker concentrations. These reagents were optimized for use in Luminex xMap technology (Alere, San Diego) or micro-titer plate ELISAs. Once optimized for format, a small subset of clinical patient samples were used to determine the final reagents (pairings or single recombinant Fabs) to use in the full cohort screening.

Luminex assays utilize magnetic beads that have a unique ratio of two dyes that are used to distinguish the identity of each bead, thereby enabling the multiplexing of assays within the same well. The Luminex sandwich assay format uses a recombinant Fab conjugated to a magnetic bead as the capture and a biotin-conjugated recombinant Fab as the assay detection. Recombinant Fab conjugated magnetic beads are added to the plate and washed. Sample is then incubated with the beads, followed by incubation with the detection antibody. The plate is washed, incubated with strepavidin labeled phycoerythrin, washed and then read using a Luminex 200 reader. The Luminex competitive assay format uses a recombinant Fab conjugated to the bead and a biotin-conjugated antigen as the assay detection. Recombinant Fab conjugated magnetic beads are added to the plate and washed. The sample and detection reagent are premixed, added simultaneously and then incubated. The final steps are as described for the Luminex sandwich assay.

The Luminex multiplex method was tested for possible interference between the measured biomarkers. If the assay did not perform in a plex format for interference it was removed from the plex and further optimized into another plex or on its own in a single assay format. Each assay was measured in a linear range.The ranges of each assay was noted and if the sample fell out of range it was clipped at the tails.

The micro-titer ELISA assays use a streptavidin coated plate and biotin or fluorescein conjugated recombinant Fabs. The ELISA sandwich assay uses a biotin-conjugated recombinant Fab as the capture and a fluorescein-conjugated recombinant Fab as the detection antibody. Capture antibody is coated on the plate, incubated, washed and sample added. After sample incubation, the plate is washed and then incubated with detection antibody. Following washing, the plate is incubated with anti fluorescein antibody conjugated to alkaline phosphatase, washed, fluorescent substrate added and then read using a Tecan infinite F200 reader.

The ELISA competitive assay uses a biotin-conjugated antigen as the capture and a fluorescein-conjugated recombinant Fab as the detection antibody. The plate is coated with capture antibody, incubated and washed. Addition of sample is immediately followed by addition of the detection antibody and incubated. The final steps are the same as the ELISA sandwich.

Each assay uses an 8 point dose curve prepared gravimetrically in EDTA plasma or buffer.

**References**

1. Fyfe EM, Rivers KS, Thompson JM, Thiyagarajan KP, Groom KM, Dekker GA, et al. Elevated maternal lipids in early pregnancy are not associated with risk of intrapartum caesarean in overweight and obese nulliparous women. BMC Pregnancy Childbirth. 2013;13:143.

2. Kenny LC, Black MA, Poston L, Taylor R, Myers JE, Baker PN, et al. Early pregnancy prediction of preeclampsia in nulliparous women, combining clinical risk and biomarkers: the Screening for Pregnancy Endpoints (SCOPE) international cohort study. Hypertens. 2014;64(3):644-52.

**Table A. Variables available to SGA, Normotensive SGA or Hypertensive SGA models**

| **Variable Name** | **Variable categories** | **Comments** | **Missing** | **Imputation type** | **All-SGA**  **N=38** | **Normotensive-SGA**  **N=26** | **Hypertensive-SGA**  **N=17** |
| --- | --- | --- | --- | --- | --- | --- | --- |
| Maternal age (years) | 1: <25y  2: 25-34y  3: ≥ 35y |  | Nil |  | ✓ | ✓ |  |
| Currently attending university | No Yes |  | Nil |  | ✓ | ✓ |  |
| Paid employment | No Yes | Included full and part-time paid employment at 15 weeks. | Nil |  | ✓ | ✓ |  |
| Participant’s birthweight | 1: <3000  2: 3000 to 3499  3: >3500 | Data was self-reported, but women were asked to bring their ‘birth record’ if kept at home. Birthweight was confirmed from this birth record in 77%. Imputed with expectation maximum likelihood estimation (EM). | 5.1% | EM | ✓ | ✓ | ✓ |
| Primigravida | No Yes |  | Nil |  | ✓ |  |  |
| Months of sexual relationship with partner ≤3 months duration. | No Yes |  | 0.2% | Median | ✓ | ✓ |  |
| Any pregnancy loss <10weeks with same partner | No Yes | Any previous termination or miscarriage <=10wks gestation or an ectopic pregnancy with same man who has fathered the current pregnancy. | Nil |  | ✓ |  |  |
| History of infertility | No Yes | History of infertility defined as >=12 months of regular intercourse without contraception and conception has not occurred or if partner is known to be sterile. 0.2% were unknown and were included in the ‘No’ category. | Nil |  | ✓ |  | ✓ |
| Hypertension on combined oral contraception (OC) | No Yes | Participant had been told on more than one occasion that her blood pressure was elevated by her health care worker when on combined OC or had ceased the OC because of raised blood pressure. | Nil |  | ✓ |  | ✓ |
| Participant’s mother had a history of gestational hypertension | No Yes | Mother of woman developed gestational hypertension defined as new onset hypertension in second half of pregnancy and known to not have proteinuria. | Nil |  | ✓ |  |  |
| Participant’s mother had a history of preterm birth | No Yes | Participant's mother had a history of recurrent preterm births (included both spontaneous and iatrogenic preterm births) | Nil |  | ✓ |  |  |
| Participant’s mother had a history of low birth weight baby | No  Yes | Mother of woman had one or more low birth weight babies defined as <2500g. | Nil |  | ✓ | ✓ |  |
| Participant’s mother had a history of metabolic disease | No  Yes | Mother of woman had one or more of type 2 diabetes, chronic hypertension, CVA and IHD. | Nil |  | ✓ |  | ✓ |
| Participant’s father has coronary heart disease | No  Yes | Father of woman has had a heart attack, coronary heart disease, coronary bypass, angioplasty or angina. If father had coronary heart disease=Yes; no paternal history of coronary heart disease or no paternal history available=No. | Nil |  | ✓ |  | ✓ |
| Vegetarian | No  Yes | Woman’s diet did not include meat or fish. | Nil |  | ✓ |  | ✓ |
| Non-oily fish intake pre-pregnancy | No  Yes | History of eating any non-oily fish including shellfish in the month prior to pregnancy | Nil |  | ✓ |  |  |
| Fruit intake pre-pregnancy | 1: <1/day  2: >1/day | The average number of servings of fruit in the month prior to conception. | Nil |  | ✓ | ✓ | ✓ |
| Fruit intake at 15 weeks | 1: <1/wk  2: 1-6x/wk  3: >=1/day | The average number of servings of fruit in the month prior to the 15 week visit. | Nil |  | ✓ |  | ✓ |
| Green leafy vegetable intake pre-pregnancy | 1: <1/wk 2: 1-6x/wk  3: >=1/day | The average number of servings of green leafy vegetables in the month prior to conception. | Nil |  | ✓ | ✓ |  |
| High oily fish at 15 weeks | No  Yes | History of eating high amounts of oily fish (defined as three or more servings per week) in the month prior to the 15 week visit. Included salmon, tuna, trout, sardines, herrings, mackerel. | Nil |  |  | ✓ |  |
| Multivitamin intake at 15±1 weeks | No  Yes | Any multivitamin intake at the 15±1 week visit. Women were asked to bring these to the 15 week visit. | 0.3% | Mode | ✓ | ✓ |  |
| Smoking at 15±1 weeks | No  Yes | Women who continued to smoke at the time of the15 week visit. | Nil |  | ✓ | ✓ |  |
| Alcohol intake at 15±1 weeks | 1: No alcohol in pregnancy  2: Quit alcohol prior to 15 weeks  3: Continuing to drink alcohol at 15 weeks | Alcohol consumption at 15 weeks included any consumption of alcohol in the week prior to the 15 week visit. | Nil |  |  | ✓ |  |
| Binge Alcohol in pregnancy | No Yes | Any binge alcohol consumed in pregnancy up to the time of the 15±1 weeks. Binge defined as the consumption of > 6 units of alcohol at one time point. | Nil |  | ✓ |  | ✓ |
| Other drug use at 15±1 weeks | No Yes | Consumed/inhaled/injected recreational drugs other than cigarettes and alcohol. Included binge drinking. | Nil |  | ✓ | ✓ |  |
| Systolic blood pressure at 15±1 weeks | 1: < 120  2: >120 | Second measurement of systolic blood pressure. GAMs in SAS was used to determine the shape of the relationship with SGA as opposed to assuming a linear relationship and 2 categories were then created. | Nil |  | ✓ |  | ✓ |
| Diastolic blood pressure at 15±1 weeks | 1: < 80  2: >80 | Second measurement of diastolic blood pressure. GAMs in SAS was used to determine the shape of the relationship with SGA as opposed to assuming a linear relationship and 2 categories were then created. | Nil |  | ✓ |  | ✓ |
| Body mass index (BMI) at 15±1 weeks | 1: <20  2: 20-24.9  3: 25-29.9  4: >30 | weight (kg) / height^2^ (m). GAMs in SAS was used to determine the shape of the relationship with SGA as opposed to assuming a linear relationship and 4 categories were then created. | Nil |  | ✓ | ✓ | ✓ |
| Head circumference at 15±1 weeks | 1: < 80  2: >80 | Maternal head circumference measured, in centimetres, GAMs in SAS was used to determine the shape of the relationship with SGA as opposed to assuming a linear relationship and 2 categories were then created. Imputed with expectation maximum likelihood estimation (EM). | 0.2% | EM | ✓ | ✓ |  |
| Random glucose at 15±1 weeks | Continuous | mmol/L; imputed with expectation maximum likelihood estimation (EM).. | 1.3% | EM | ✓ | ✓ |  |
| Proteinuria at 15±1 weeks | No Yes | Defined as 1+ on dipstick or spot urine protein creatinine ratio (PCR) measurement ≥30 mg/mmol. | 0.6% | Mode dipstick  Median PCR | ✓ | ✓ | ✓ |
| Recreational walking at 15±1 weeks | <4 x /wk  >4 x /wk | Engaged in any walking for recreation or exercise in the last month. | 0.4% | Mode | ✓ | ✓ |  |
| Vigorous exercise at 15±1 weeks | No Yes | Engaged in any daily exercise leading to heavy breathing or being puffed. | 0.4% | Mode | ✓ | ✓ |  |
| Rhesus factor negative | No Yes |  | Nil |  | ✓ | ✓ |  |
| Head circumference z score <10^th^ centile at 20 week scan | No Yes | Fetal head circumference (adjusted for gestational age using multiples of the median) <10^th^centile |  |  | ✓ | ✓ | ✓ |
| Abdominal circumference z score <10^th^ centile at 20 week scan | No Yes | Fetal abdominal circumference (adjusted for gestational age using multiples of the median) <10^th^centile |  |  | ✓ | ✓ |  |
| Femur length z score <10^th^ centile at 20 week scan | No Yes | Fetal femur length (adjusted for gestational age using multiples of the median) <10^th^centile |  |  | ✓ | ✓ |  |
| Umbilical artery resistance index (RI) at 20 week scan | 1: <0.7  2: 0.7-0.79  3: 0.8-1.0 | Umbilical artery Resistance Index (RI) measured using Doppler ultrasound at 19-21w 3 categories created. | Nil |  | ✓ | ✓ | ✓ |
| Mean uterine artery RI at 20 week scan | 1: <0.5  2: 0.5 to 0.59  3: 0.6 to 0.69  4: 0.7 to 0.79  5: 0.8 to 1.0 | Mean uterine RI was calculated from the right and left uterine artery RI. Five categories were created for All-SGA and Hypertensive-SGA and four categories (<0.5/0.5 to 0.59/0.6 to 0.69/0.7 to 1.0) for Normotensive-SGA. | Nil |  | ✓ | ✓ | ✓ |
| Bilateral notches at 20 week scan | No Yes | Diastolic notches in the Doppler waveforms of both the right and left uterine arteries. Reference notch photos provided to ultrasonographers. | Nil |  | ✓ | ✓ | ✓ |

**Table B. List of biomarkers measured at 14-16 weeks’ gestation and the assay method.**

| **Biomarker** | **Assay method** | **Included in analysis** | **Convert to MoM** |
| --- | --- | --- | --- |
| Adam-9 (Disintegrin and metalloproteinase domain-containing protein 9) | Luminex Sandwich | Exclude LOD ^a^ |  |
| Adiponectin ^b^ | Luminex Competitive | Yes |  |
| Angiogenin | Luminex Competitive | Yes |  |
| Arginase-1 | Luminex Sandwich | Yes |  |
| Arginase-2 | Luminex Sandwich | Yes |  |
| Atrial natriuretic peptide (ANP)-propeptide | Luminex Sandwich | Yes |  |
| Big Endothelin-1 | Luminex Sandwich | Exclude LOD ^a^ |  |
| Brain natriuretic peptide (BNP) | Luminex Sandwich | Yes | Yes |
| C-Met | Luminex Sandwich | Yes |  |
| C-reactive protein (CRP) | Luminex Competitive | Yes |  |
| C-X-C motif chemokine 10 (CXCL 10) | Luminex Sandwich | Yes |  |
| Carboxypeptidase A4 (CPA-4) precursor | Luminex Sandwich | Yes |  |
| Caspase-3 | Luminex Sandwich | Yes |  |
| Chemokine (C-C motif) ligand 23 (CCL23) | Luminex Sandwich | Yes |  |
| Cholesterol (total) ^b^ | Enzymatic Colorimetric | Yes | Yes |
| Cystatin C | ELISA Competitive | Yes |  |
| Elafin | Luminex Competitive | Yes |  |
| Endoglin | ELISA Sandwich | Yes |  |
| Endothelial cell-selective adhesion molecule (ESAM-1) | Luminex Sandwich | Yes |  |
| Ephrin-receptor-2 | Luminex Sandwich | Exclude LOD ^a^ |  |
| Factor inhibiting hypoxia inducible factor 1α (FIH) | Luminex Sandwich | Yes |  |
| Fas cell surface death receptor (FAS) | Luminex Sandwich | Yes | Yes |
| Fas ligand (Fas L)-soluble | Luminex Sandwich | Exclude LOD ^a^ |  |
| HDL-cholesterol ^b^ | Enzymatic Colorimetric | Yes |  |
| Insulin ^b^ | Luminex Sandwich | Yes |  |
| Intercellular adhesion molecule-1 (ICAM-1) | Luminex Competitive | Yes |  |
| Interleukin 1 receptor antagonist (IL-1ra) | Luminex Sandwich | Yes |  |
| Kunitz type protease inhibitor 2 (HAI-2) | Luminex Sandwich | Yes |  |
| LDL-cholesterol ^b^ | Calculated (Friedewald Formula) | Yes | Yes |
| Leptin | Luminex Sandwich | Exclude  LOD ^a^ |  |
| Leptin receptor | Luminex Sandwich | Yes |  |
| Macrophage migration inhibitory factor (MIF) | Luminex Sandwich | Yes |  |
| Matrix metalloproteinase-9 (MMP-9) | Luminex Sandwich | Yes |  |
| Nephrin | Luminex Sandwich | Exclude LOD ^a^ | Yes |
| Neutrophil gelatinase-associated lipocalin (NGAL) | ELISA Sandwich | Yes |  |
| Pentraxin-3 | Luminex Sandwich | Exclude LOD ^a^ |  |
| Periostin | Luminex Sandwich | Yes |  |
| Placental growth factor (PlGF) | Luminex Sandwich | Yes | Yes |
| Placental growth hormone (PlGH) ^b^ | Luminex Sandwich | Yes | Yes |
| Plasminogen activator inhibitor 1 (PAI-1) | Luminex Sandwich | Exclude LOD ^a^ |  |
| Plasminogen activator inhibitor 2 (PAI-2) | Luminex Sandwich | Yes | Yes |
| Podocalyxin | Luminex Sandwich | Exclude LOD ^a^ |  |
| Pregnancy associated plasma protein A (PAPP-A) | Luminex Sandwich | Yes | Yes |
| Procalcitonin (PCT) | Luminex Sandwich | Exclude LOD ^a^ |  |
| ST2 | Luminex Sandwich | Yes |  |
| TIMP metallopeptidase inhibitor 1 (TIMP-1) | Luminex Competitive | Yes |  |
| Transforming growth factor (TGF) β receptor 2 | Luminex Sandwich | Yes |  |
| Triglycerides ^b^ | Enzymatic Colorimetric | Yes | Yes |
| Tumour necrosis factor receptor 1a (TNFR1a) | Luminex Sandwich | Yes |  |
| Vascular endothelial growth factor C (VEGF-C) | Luminex Sandwich | Yes |  |
| Vascular endothelial growth factor receptor 1 (VEGFR1) | Luminex Sandwich | Yes |  |
| Visfatin | Luminex Sandwich | Exclude LOD ^a^ |  |
| WAP four disulfide core domain protein 2 (HE4) | Luminex Sandwich | Yes |  |

^a^ Excluded as the majority of measurements for the biomarker were below the limit of detection (LOD) of the assay.

^b^ Biomarkers previously reported in Kenny et al^2^ except adiponectin, cholesterol (total), HDL-cholesterol insulin, LDL-cholesterol, PlGH, triglycerides.
